# Patchoulene epoxide mitigates colitis and hepatic damage induced by dextran sulfate sodium by regulating the colonic microbiota and purine metabolism

**DOI:** 10.3389/fimmu.2025.1509114

**Published:** 2025-02-14

**Authors:** Liping Chen, Lili Xie, Lifen Wang, Xueli Zhan, Zhenjian Zhuo, Susu Jiang, Lei Miao, Xinxin Zhang, Weiming Zheng, Tzu-Ming Liu, Jing He, Yuhong Liu

**Affiliations:** ^1^ Department of Pediatric Surgery, Guangzhou Institute of Pediatrics, Guangdong Provincial Key Laboratory of Research in Structural Birth Defect Disease, Guangzhou Women and Children’s Medical Center, Guangzhou Medical University, Guangzhou, Guangdong, China; ^2^ Cancer Center, Faculty of Health Sciences, University of Macau, Macau, Macao SAR, China; ^3^ Laboratory Animal Center, School of Chemical Biology and Biotechnology, Peking University Shenzhen Graduate School, Shenzhen, China; ^4^ Institute of Translational Medicine, Faculty of Health Sciences & Ministry of Education Frontiers Science Center for Precision Oncology, University of Macau, Taipa, Macau, China; ^5^ School of Pharmaceutical Sciences, Guangzhou University of Chinese Medicine, Guangzhou, China

**Keywords:** patchoulene epoxide, ulcerative colitis, liver injury, colonic microbiota, purine metabolism

## Abstract

**Introduction:**

Ulcerative colitis (UC) is often characterized by dysbiosis of the colonic microbiota and metabolic disturbances, which can lead to liver damage. Patchoulene epoxide (PAO), a tricyclic sesquiterpene derived from the aged essential oil of Pogostemonis Herba, is known for its anti-inflammatory and ulcer-healing properties. However, its dual protective role against UC and liver injury remains largely unexplored. This study aims to elucidate the protective effect and underlying mechanism of PAO against dextran sulfate sodium (DSS)-induced UC and liver injury in mice.

**Methods:**

Colitis and liver injury in mice were induced by adding 3% DSS to their drinking water continuously for 7 days, and PAO at the doses of 20 and 40 mg/kg was administered orally to mice daily from the first day until the experimental endpoint. Stool consistency scores, blood stool scores, and body weights were recorded weekly. Disease activity index (DAI) was determined before necropsy, where colon and liver tissues were collected for biochemical analyses. Additionally, the fecal microbiome and its metabolites of treated mice were characterized using 16S rRNA amplicon sequencing and metabolomics.

**Results:**

PAO significantly reduced the disease activity index and mitigated colonic atrophy in UC mice. It also improved colonic and hepatic pathological changes by safeguarding tight and adherens junctions, and suppressing the generation of pro-inflammatory cytokines and lipopolysaccharide. These beneficial effects were attributed to PAO’s capability to regulate the colonic microbiota and metabolic processes. PAO was found to enhance the diversity of the colonic microbiota and to shift the microbial balance in UC mice. Specifically, it restored the microbiota from an Akkermansia-dominated state, characteristic of UC, to a healthier Muribaculaceae-dominated composition. Furthermore, PAO corrected the colon metabolic disturbance in UC mice by modulating the purine metabolism, notably increasing the abundance of deoxyadenosine, adenosine and guanine in UC mice.

**Conclusions:**

The therapeutic effect of PAO on UC and liver injury was mainly attributed to its regulation of colonic microbiota and purine metabolism. These insights emphasize the overall therapeutic benefits of PAO in treating UC and liver injury.

## Introduction

Ulcerative colitis (UC), a form of inflammatory bowel disease, is characterized by persistent inflammation and ulceration within the colonic mucosa. This condition presents a range of clinical symptoms, including abdominal pain, diarrhea, and bloody stools (1). The prevalence of UC is notably high and continues to rise globally, particularly in industrialized Western countries (2). Although mesalazine, corticosteroids and immunosuppressants are standard treatments for UC, they fall short of providing a complete cure and can cause adverse effects with long-term use (3, 4). Therefore, there is a pressing need to identify more effective therapeutic agents and pathways for the management of UC.

The pathogenesis of UC is complex and multifactorial, with studies highlighting the critical roles of dysbiosis in the colonic microbiota and metabolic disorders in the disease’s development (5, 6). Among patients with UC, these imbalances frequently result in an overgrowth of pathogenic bacteria, which not only impair the integrity of the colonic barrier but also amplify the inflammatory response, thus worsening the disease state (7). This breach in the colonic barrier function escalates intestinal permeability, precipitating leakage into the gut and the subsequent translocation of bacterial endotoxins and pro-inflammatory cytokines to the liver, leading to hepatic injury (8, 9). Consequently, this liver damage provokes the emission of more pro-inflammatory cytokines, perpetuating and intensifying the progression of UC (10). This complex interplay between the gut and liver highlights the importance that a comprehensive approach to UC treatment should also address liver injury. For instance, berberine has been shown to alleviate UC by reducing pathogenic bacterial colonization and improving bile acid metabolism via the gut-liver axis (11). Emerging evidence also highlights the role of fecal microbiota transplantation in re-establishing a healthy gut-liver axis, with studies showing improvements in both intestinal and hepatic biomarkers of inflammation (12). Taken together, these findings support the hypothesis that preserving the colonic barrier through modulation of gut microbiota and metabolism could offer a promising strategy for treating both UC and liver damage.

**Figure 1:**
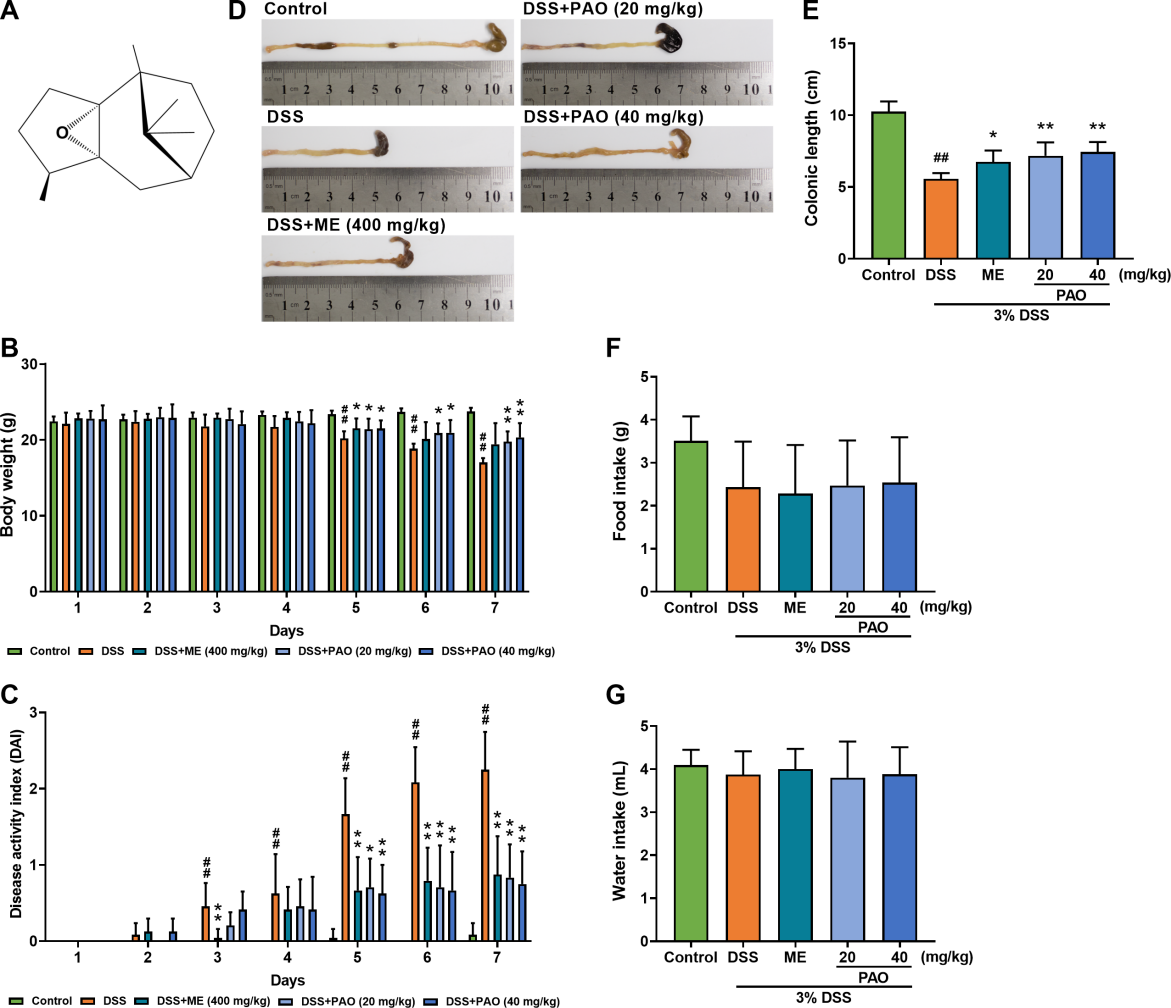
Effect of PAO on disease activity index (DAI) and colonic length in UC mice. **(A)** Chemical structure of PAO. **(B)** Body weight changes. **(C)** DAI assessment. **(D)** Representative macroscopic images of colons. **(E)** Colonic length. **(F)** Food and **(G)** Water intake. Data are presented as means ± SD (*n*=8). ^#^ *P*<0.05, ^##^ *P*<0.01, vs. Control group; ^*^ *P*<0.05, ^**^ *P*<0.01, vs. DSS group.

*Pogostemon cablin* (Blanco) Benth. (Labiatae), commonly known as Pogostemonis Herba or “Guang-Huo-Xiang” in traditional Chinese medicine, is widely used in China for the treatment of gastrointestinal disorders (13). Patchoulene epoxide (PAO, **Figure 1A**), a tricyclic sesquiterpene derived from the aged essential oil of Pogostemonis Herba (14), has been reported to possess anti-inflammatory (14) and anti-ulcer (15) properties. The chemical structure of PAO closely resembles that of patchouli alcohol and patchoulene, which are the active constituents of Pogostemonis Herba, traditionally used for the treatment of gastrointestinal disorders (14, 15). Patchouli alcohol and patchoulene have been documented for their superior anti-UC (8, 16) and hepatoprotective effects (8, 17–20). It is hypothesized that PAO, as an epoxide derivative of patchouli alcohol and patchoulene, may also possess similar therapeutic properties against UC and offer hepatoprotection. However, the potential of PAO in mitigating UC and liver damage has yet to be elucidated. Given the significance of the gut-liver axis in UC treatment and the crucial role of colonic flora and metabolism in maintaining barrier function, we have embarked on a study to explore the protective effects of PAO and its possible mechanisms against dextran sulfate sodium (DSS)-induced colitis and liver injury in mice, focusing on the modulation of the colonic microbiota and metabolism.

## Materials and methods

### Reagents

Patchoulene epoxide (PAO, PubChem CID: 14164179, with a purity exceeding 95%, **Figure 1A**) was obtained following our previous study (14). Dextran sulfate sodium (DSS) was procured from MP Biomedical, LLC, France. Mesalazine (ME) was acquired from Pharmaceutical Ltd, Lausanne, Germany. All reagents used were of the analytical grade.

### Experimental animals

Healthy male BALB/c mice (21~25 g) were sourced from the Laboratory Animal Management Center of Southern Medical University (Guangzhou, China, License No.44002100026515). Mice were housed under standard environmental conditions (temperature maintained at 23 ± 2 ℃, 60 to 70% humidity, and a 12-hour light/dark cycle), with ad libitum access to food and distilled water. All animal procedures were strictly carried out in accordance with the guidelines approved by the Ethics Committee for Animal Experiments of Guangzhou University of Chinese Medicine (Approval No.00248761).

### Induction of colitis and liver injury, and PAO treatment

After a one-week acclimatization period, the mice were randomly assigned to five groups (*n*=12 each), including Control group, DSS group, DSS with ME (400 mg/kg) group, and two DSS with PAO groups (20 and 40mg/kg). All mice were provided with water containing 3% (w/v) DSS for 7 days with a daily DSS intake of 6g/kg body weight to induce colitis and liver injury except for the mice of Control group (8). Concurrently, the Control and DSS groups were administered a 1% (w/v) Tween-80 water solution orally, while the remaining groups received the respective drugs dissolved in the same Tween-80 solution. The mice were orally administered once daily. The dosage of PAO and ME was determined based on prior experimental data (14, 15, 21). Body weight, fecal traits, rectal bleeding, food and water intake of all mice were recorded daily. Upon completion of the experiment, the mice were anesthetized for blood collection and subsequently euthanized to harvest the colon, its contents, and the liver for further analysis.

### Disease activity index assessment

Body weight, fecal characteristics, and rectal bleeding of mice were recorded daily throughout the study. Weight loss, fecal traits and rectal bleeding scores were assessed by an independent observer blinded to the study details using a standardized scoring system (22) (**Table 1**). The disease activity index (DAI) was calculated by averaging these individual scores, resulting in a scale from 0 (indicating normalcy) to 4 (representing severe colitis activity).

**Table 1:** Disease activity index (DAI) score.

Weight lose/%	Stool consistency	Rectal bleeding	Grade
0	Normal	None	0
1-5	Soft	Mild bleeding	1
5-10	Soft and wet	Moderate bleeding	2
10-20	Half loose stool	Heavy bleeding	3
>20	Loose stool	Blood clots around the anus	4

### Histopathological examination

Colon and liver tissues were fixed using a 4% paraformaldehyde solution. Following dehydration, the tissues were embedded in paraffin, sectioned, and stained with hematoxylin and eosin (H&E). Pathological evaluation was conducted under a light microscope by a pathologist blinded to this study following a predefined scoring system (23–25) (**Tables 2**, **3**).

**Table 2:** Colonic histopathological score.

Severity of inflammation	Extent of ulcerative	Crypt damage	Grade
None	None	None	0
Mild	Mucosa	1/3 damaged	1
Moderate	Mucosa and submucosa	2/3 damaged	2
Severe	Transmural	Only epithelium was left intact	3
–	–	Both crypt and epithelium lost	4

**Table 3:** Liver histopathological score.

Hepatocyte ballooning	Lobular inflammation	Grade
None	None	0
Scattered balloon cells	**<**2 foci**/×**200 field	1
Panacinar balloon cells	2 to 4 foci**/×**200 field	2
–	**>**4 foci**/×**200 field	3

### Analysis of liver enzymes, lipopolysaccharides and inflammatory cytokines

Serum levels of aspartate aminotransferase (AST) and alanine aminotransferase (ALT) were measured following the manufacturer′s instructions (Jiancheng Company, Nanjing, China). The concentrations of serum lipopolysaccharide (LPS), colonic LPS and inflammatory cytokines (TNF-α, IL-1β and IL-6), as well as hepatic LPS and inflammatory cytokines (TNF-α, IL-1β and IL-6) were determined following the manufacturer′s protocol (Enzyme-linked Biotechnology Co., Ltd., Shanghai, China).

### Quantitative real-time polymerase chain reaction analysis

Total RNA was extracted from the harvested colon tissue using Trizol reagent, following the manufacturer’s guidelines. Subsequently, this RNA was reverse-transcribed into complementary DNA (cDNA) using the reverse transcription reagent (Vazyme, Shanghai, China). The cDNA samples were then subjected to qRT-PCR analysis with primers (Biotechnology Biotechnology Co., Ltd. Shanghai, China), 2×ChamQ SYBR qPCR Master Mix (Vazyme, Shanghai, China), and managed by the CFX Manager software (Bio-Rad Laboratories Inc.). The qRT-PCR conditions were optimized with precycling at 95°C for 30 s, followed by 39 cycles at 95°C for 10 s and 60°C for 30 s, then 95°C for 15 s, 65°C for 5 s, and Melt Curve 65°C to 95°C increments 0.5°C. Relative genes expressions of α-catenin, β-catenin, Claudin-1 and ZO-1 were determined using the 2^−ΔΔCt^ method. The primer sequences utilized in this study are detailed in **Table 4**.

**Table 4:** Primers for quantitative real-time PCR.

Gene	Primer Sequences
α-catenin	Forward: 5′-GATAAAACCCTGCAAGCGATTC-3′ Reverse: 5′-ATCCCAGTTTTCGTTGGCTATA-3′ Forward: 5′-CTGCTGTCCTATTCCGAATGTCTGAG-3′ Reverse: 5′-GGCACCAATGTCCAGTCCAAGATC-3′ Forward: 5′-CTGGTGAAGTCTCGGAAAAATG-3′ Reverse: 5′-CATCTCTTGCTGCCAAACTATC-3′ Forward: 5′-AGATACAGTGCAAAGTCTTCGA-3′ Reverse: 5′-CAGGATGCCAATTACCATCAAG-3′ Forward: 5′-CTACCTCATGAAGATCCTGACC-3′ Reverse: 5′-CACAGCTTCTCTTTGATGTCAC-3′
β-catenin	
ZO-1	
Claudin-1	
β-actin	

### Immunohistochemistry of ZO-1 and β-catenin

Colon paraffin sections were placed in an oven at 60°C for 3 h and dewaxed with different concentrations of xylene. Then they were placed in 3% H_2_O_2_ to block endogenous peroxidase and sealed with 3% BSA. Sections were incubated with primary antibody ZO-1 and β-catenin (all 1: 1000) at 4°C overnight and secondary antibody at indoor temperature for 90 min. Subsequently, the sections were then stained with DAB and hematoxylin, dehydrated, and sealed. All liver sections were observed under a microscope and analyzed using Image-Pro Plus 6.0 (Media Cybernetics, Inc.).

### 16S rDNA gene sequencing

Genomic DNA from colonic contents was extracted using the E.Z.N.A.^®^ Stool DNA Kit (Omega Bio-Tek, USA) following the manufacturer′s protocol. The V3-V4 region of the bacterial 16S rDNA gene was amplified with specific primers: forward primer 5′-ACTCCTACGGGAGGCAGCAG-3′ and reverse primer 5′-GGACTACHV GGGTWTCTAAT-3′. PCR products were purified using AMPure XT beads (Beckman Coulter Genomics, Danvers, MA, USA) and quantified with Qubit fluorometer (Invitrogen, USA). The qualified libraries were sequenced pair end on the HiSeq System (HiSeq SBS Kit V2, Illumina) using NovaSeq 6000 Sequencer.

### Bioinformatics data analysis

Sequencing yielded double-ended data was processed to remove the joint and barcode sequences. FLASH (version 1.2.11) was employed to merge compatible end reads. The raw data was filtered under stringent conditions to generate high-quality clean tags using fqtrim (v 0.94), and chimeric sequences were identified and removed with Vsearch (v2.3.4). ASV (feature) sequence and ASV (feature) abundance tables were generated through length filtering and denoising with QIIME2 dada2 denoise-paired. Diversity indices were calculated by normalizing to an equal number of sequences per sample. Feature abundance was normalized relative to each sample according to the SILVA (release 132) classifier. Alpha diversity analysis, including observed_species, shannon, and chao1 indices, was conducted with QIIME2. Beta diversity analysis was performed with QIIME2 and visualized using the R package to assess the diversity among samples/groups with uniFrac distance-based principal coordinates analysis (PCoA). Linear discriminant analysis (LEfSe) analysis was conducted using an online tool (http://huttenhower.sph.harvard.edu/galaxy/), and microbial groups with LDA scores above 4 were considered to have significantly changed in relative abundance.

### Metabolomics analysis

Metabolites were extracted using the organic reagent protein precipitation method, and quality control (QC) samples were prepared by pooling equal amounts of experimental samples. The extracted samples were randomized and analyzed using an ultra-high-performance liquid chromatography (UPLC) system (SCIEX, UK) equipped with an ACQUITY UPLC T3 column (100 mm**×**2.1 mm, 1.8 µm, Waters, UK). QC samples were interspersed throughout the analysis to ensure technical consistency. A high-resolution tandem mass spectrometer, TripleTOF 5600plus (SCIEX, UK), was employed to detect metabolites eluting from the column. Raw data were converted to the mzXML format. Peak extraction and quality control were executed using XCMS software, and metabolites were annotated using CAMERA. Identification was performed with MetaX software, with candidate substances annotated against HMDB and KEGG databases to elucidate their physicochemical properties and biological functions. Differential metabolites were quantified and screened using MetaX software. Subsequent analyses included 3D and 2D Principal Component Analysis (PCA), Orthogonal Partial Least Squares-Discriminant Analysis (OPLS-DA), Volcano plots, Venn diagrams, and Receiver operating characteristic curve (ROC) analysis were conducted with Omicshare online tools (https://www.omicshare.com/tools/Home/Soft/getsoft/type/index). Enrichment and pathway analyses were performed using Metaboanalyst online tools (https://www.metaboanalyst.ca/MetaboAnalyst/faces/home.xhtml).

### Statistical analysis

Experimental data are expressed as the mean **±** SD. Statistical analysis was conducted using SPSS software (version 26.0), beginning with a normality test. Data conforming to a normal distribution were analyzed using one-way ANOVA, while non-parametric data were assessed using the Kruskal-Wallis test. A *P*-value of less than 0.05 was considered to indicate statistical significance. Spearman correlation analysis was employed to evaluate the relationships between various parameters.

## Results

### PAO reduced disease activity and colon shortening in UC mice

Significant weight loss was observed in the DSS group from day 5 to 7 compared to the Control group (*P*<0.05). However, treatment with PAO effectively (*P*<0.05) mitigated this weight loss starting from day 5 (**Figure 1B**). The Disease Activity Index (DAI), a critical metric for colitis severity, showed a notable (*P*<0.05) increase in the DSS group from day 3 relative to the Control group. Compared with the DSS group, treatment with PAO and ME significantly (*P* **<**0.05) lowered the DAI score from day 5 to 7 (**Figure 1C**). Colon atrophy, a common UC symptom, was evident in the DSS group with a significant (*P* **<**0.01) reduction in colon length as compared with that of the Control group. PAO treatment substantially (*P* **<**0.01) inhibited this UC-induced colon shortening (**Figures 1D, E**). No significant differences (*P* **>**0.05) were observed in food and water intake among the groups (**Figures 1F, G**).

### PAO enhanced colonic barrier function and reduced inflammation and LPS levels in UC mice

**Figure 2:**
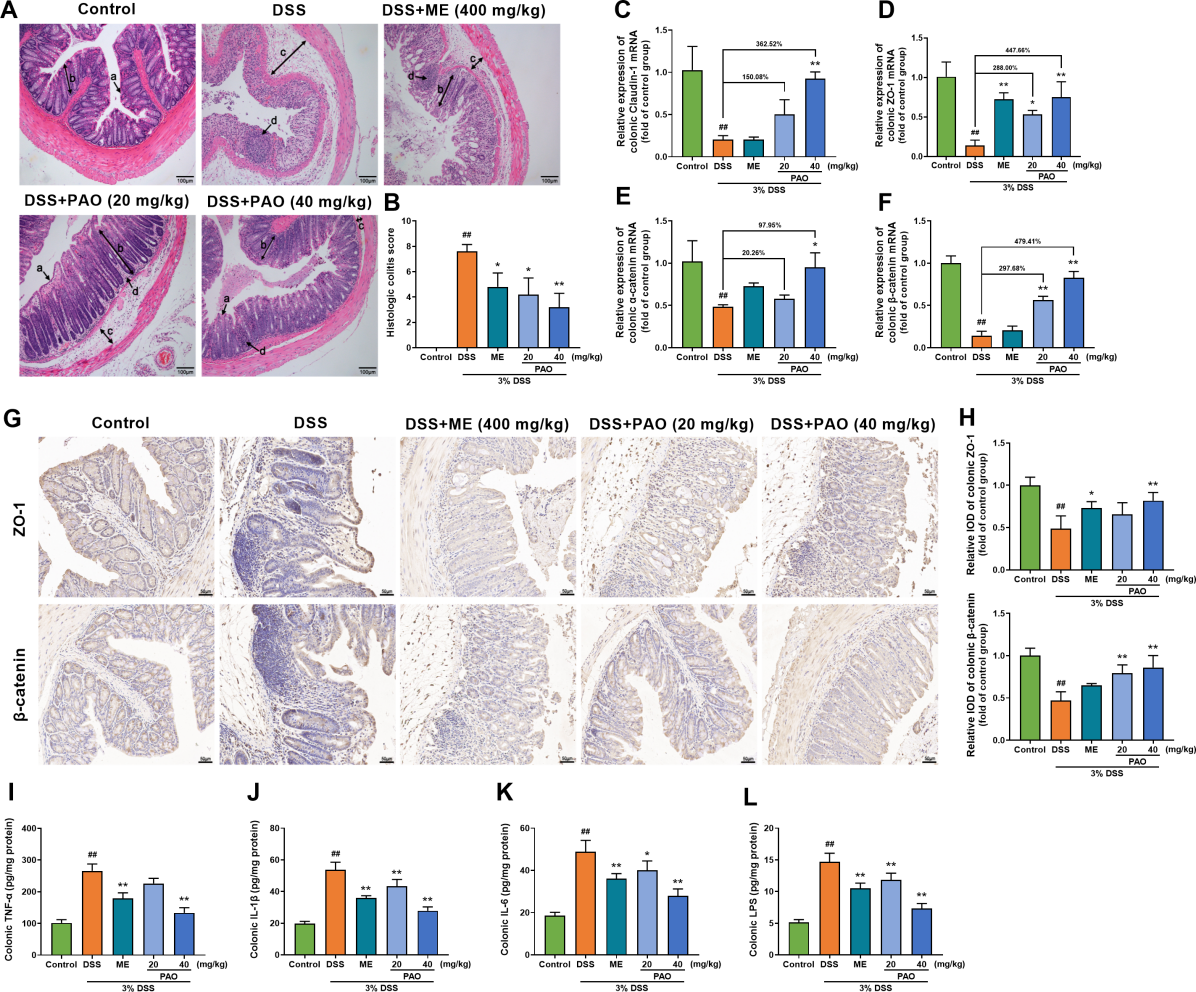
Effect of PAO on colonic histopathology, barrier integrity, proinflammatory factors and lipopolysaccharide (LPS) levels in UC mice. **(A)** H&E staining of the colon sections (magnification 100×, *n*=5). Arrows indicate (a) Intact epithelium; (b) Crypt lumen; (c) Edema; and (d) Leukocyte infiltration. **(B)** Histological scores of colonic damage. Quantitative analysis of colonic mRNA expressions for **(C)** Claudin-1, **(D)** ZO-1, **(E)** α-catenin and **(F)** β-catenin (*n*=8): The percentage represents the changing amplitude in the effect of PAO treatment as compared to the DSS group. **(G)** IHC staining of ZO-1 and β-catenin in colons (magnification 200×) and **(H)** relative integrated optical density (IOD) of colonic ZO-1 and β-catenin (*n*=5). Colonic levels of **(I)** TNF-α, **(J)** IL-1β, **(K)** IL-6 and **(L)** LPS (*n*=8). Data are presented as means ± SD. ^##^ *P*<0.01, vs. Control group; ^*^ *P*< 0.05, ^**^ *P*< 0.01, vs. DSS group.

H&E staining revealed intact colonic epithelial cells in the Control group, whereas the DSS group exhibited disrupted mucosal structure and significant inflammatory cell infiltration with a markedly (*P* **<**0.01) increased histologic score. Treatment with PAO and ME alleviated these effects, and histologic scores were significantly (*P* **<**0.05) reduced in the PAO or ME treatment group compared to the DSS group (**Figures 2A, B**).

The mRNA expressions of colonic barrier-related genes, including Claudin-1, ZO-1, α-catenin and β-catenin, were markedly (*P* **<**0.01) downregulated in the DSS group compared to the Control group. PAO treatment significantly (*P* **<**0.05) restored these expressions, except for the 20 mg/kg dose in Claudin-1 and α-catenin (**Figures 2C–F**). ME treatment markedly (*P* **<**0.01) increased mRNA expressions of ZO-1. Interestingly, among the promotional effects of PAO on colonic barrier proteins, PAO had the stronger role on regulating ZO-1 and β-catenin than those of α-catenin and Claudin-1 with amplitude change of 447.66% and 479.41%, respectively (**Figures 2C–F**). To fully demonstrate the protective effect of PAO on the colon barrier, we used immunohistochemistry to illustrate the distribution of ZO-1 and β-catenin in colonic epithelial cells. Compared with the Control group, the relative integrated optical density (IOD) of ZO-1 and β-catenin were significantly (*P*<0.01) decreased in the DSS group (**Figures 2G, H**). Treatment with PAO significantly (*P*<0.05) attenuated the reduced IOD caused by colitis. Additionally, DSS group showed significantly (*P*<0.01) higher colonic levels of TNF-α, IL-1β, IL-6, and LPS, which were substantially (*P*<0.01) reduced by PAO and ME treatments, except for the 20 mg/kg PAO treatment in TNF-α level (**Figures 2I–L**).

### PAO mitigated liver injury by reducing hepatic transaminase and inflammation

**Figure 3:**
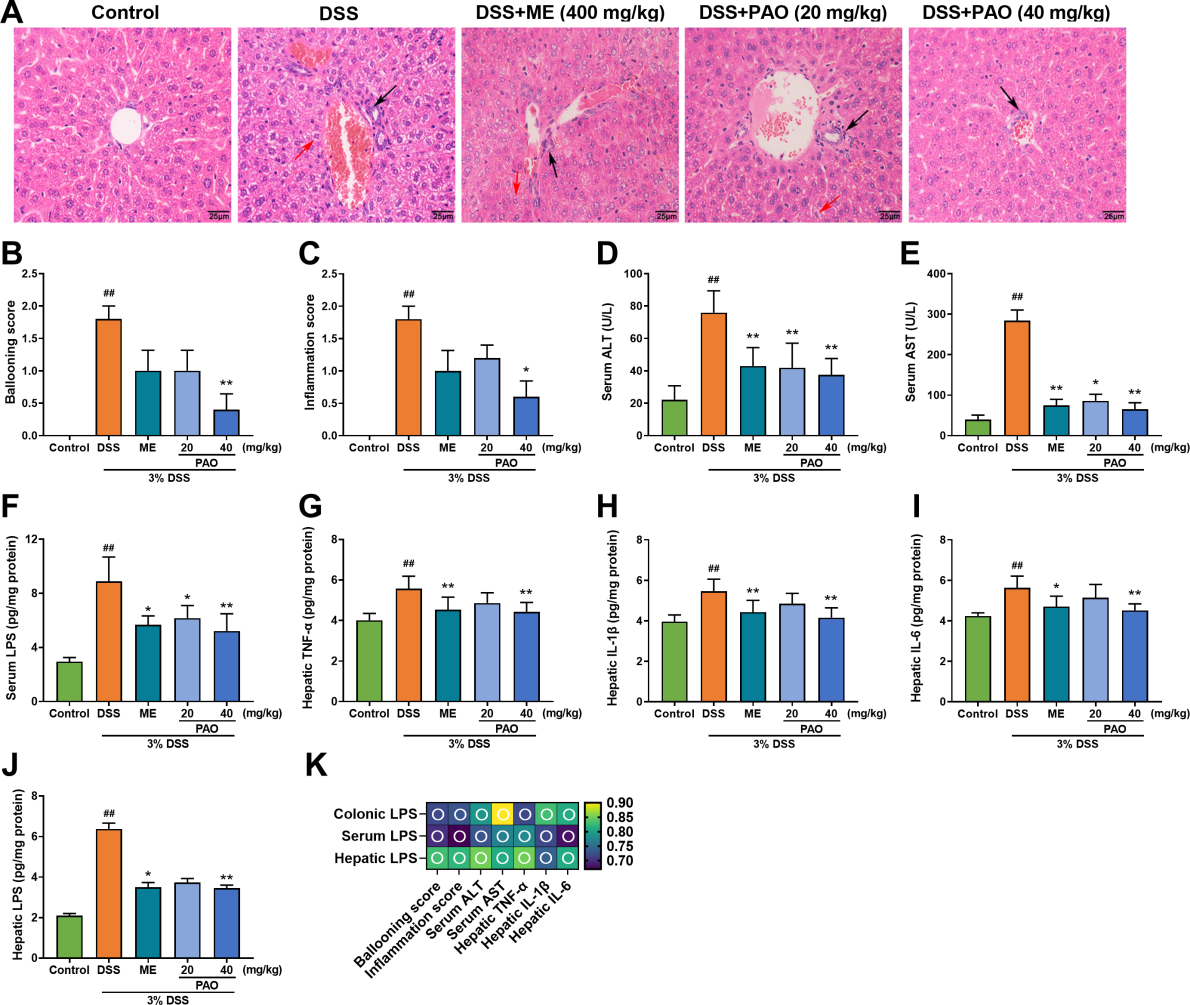
Effect of PAO on liver injury and inflammation in UC mice. **(A)** H&E staining of liver sections (magnification 400×, *n*=5), with black arrows highlighting areas of inflammation and red arrows indicating hepatocyte ballooning. **(B)** Ballooning score. **(C)** Inflammation score. Serum levels of **(D)** ALT, **(E)** AST and **(F)** LPS (*n*=8). Hepatic levels of **(G)** TNF-α, **(H)** IL-1β, **(I)** IL-6 and **(J)** LPS (*n*=8). Data are presented as means ± SD. ^##^ *P*< 0.01, vs. Control group; ^*^ *P*<0.05, ^**^ *P*<0.01, vs. DSS group. **(K)** Spearman correlation analysis presented as a heatmap, depicting the relationship between LPS and indictors of liver injury, including ballooning score, inflammation score, ALT, AST, TNF-α, IL-1β and IL-6: hollow circle indicates |r|>0.6 and *P*<0.01.

Colonic barrier disruption in colitis can lead to liver damage. H&E staining of liver sections from the Control group showed a complete structure and radially arranged liver cord, while the DSS group displayed significant lesions, hepatocyte destruction, vacuolation, and inflammation (**Figure 3A**). The pathological scores for ballooning and inflammation were remarkedly (*P* **<**0.01) higher in the DSS group in comparison to the Control group (**Figures 3B, C**). Only the 40 mg/kg PAO treatment significantly (*P* **<**0.05) reduced these elevated hepatic pathological scores caused by colitis. Compared with the Control group, serum levels of ALT, AST and LPS, as well as hepatic levels of TNF-α, IL-1β, IL-6 and LPS were markedly (*P* **<**0.01) increased in the DSS group. Treatment with 40 mg/kg PAO and ME significantly (*P* **<**0.05) attenuated these levels elevated by colitis (**Figures 3D–J**). The 20 mg/kg PAO treatment remarkedly (*P* **<**0.05) lowered serum ALT, AST and LPS levels. The portal vein serves as a crucial blood vessel connecting the gut and liver, and to confirm a direct causal link between gut-derived LPS and liver injury, a correlation analysis was conducted between LPS and indictors of liver injury, including ballooning score, inflammation score, ALT, AST, TNF-α, IL-1β and IL-6. A strong positive correlation between colonic LPS levels and liver injury indicators, including serum ALT, serum AST, and hepatic pro-inflammatory cytokines (TNF-α, IL-1β and IL-6), with most correlation coefficients exceeding 0.8 (**Figure 3K**).

### PAO improved colonic flora diversity and abundance in UC mice

**Figure 4:**
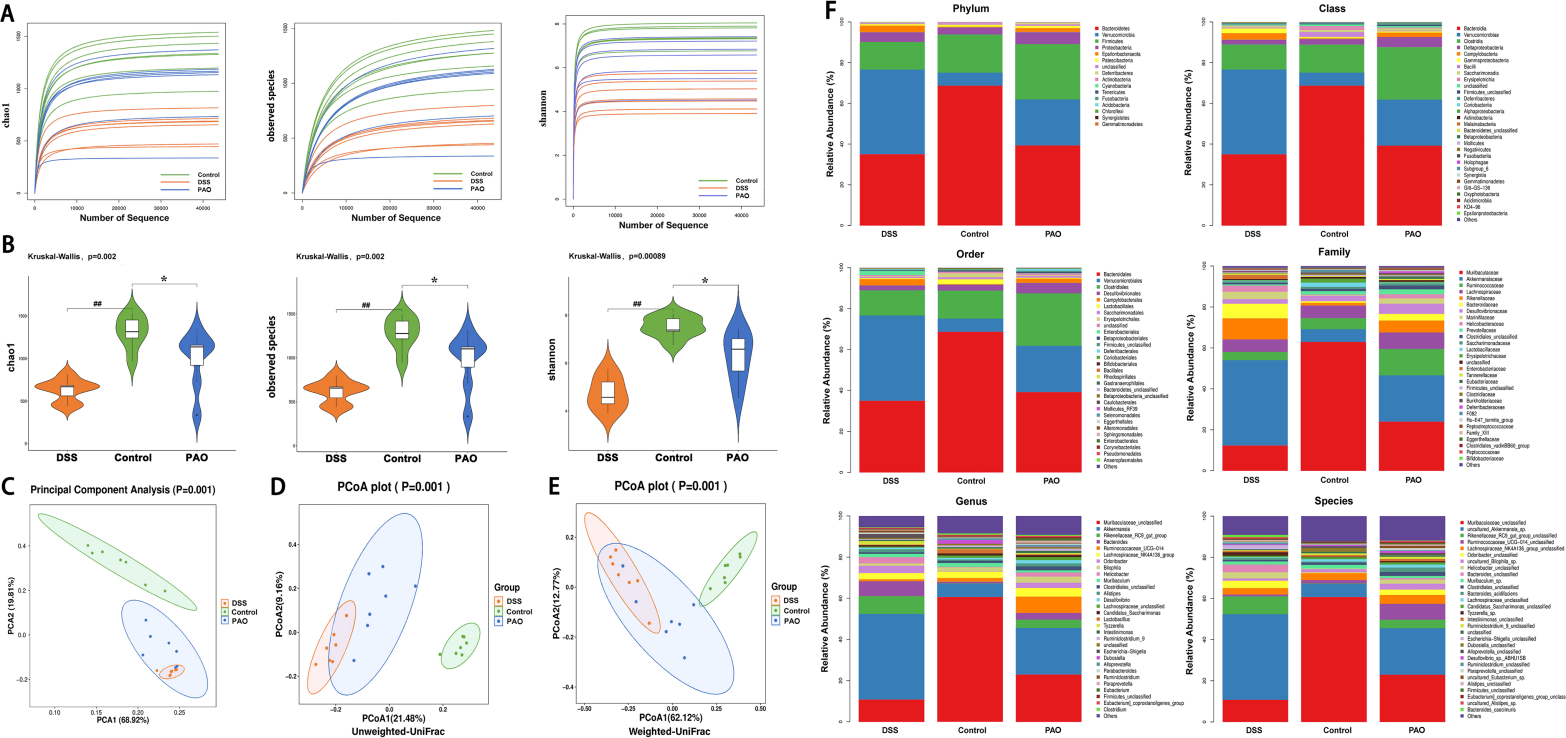
Analysis of microbial diversity and abundance (*n*=7). **(A)** Rarefaction curves depicting alpha diversity indices. **(B)** Comparison of alpha diversity indices: Statistical tests were conducted using the Kruskal-Wallis method, ^##^ *P*<0.01, vs. Control group; *P<0.05, vs. DSS group. **(C)** PCA score plot. PCoA-based beta-diversity measurements using **(D)** unweighted and **(E)** weighted UniFrac distances. **(F)** Taxonomic analysis of the colonic microbiota at phylum, class, order, family, genus, and species levels.

Colonic barrier disruption is closely linked to microbial imbalance. The plateauing of the dilution curve suggests that the sequencing depth had adequately captured the full spectrum of species present in the samples (**Figure 4A**). Alpha diversity, measured by Observed species, Chao1, and Shannon indices, was significantly (*P* **<**0.01, versus the Control group) reduced in the DSS group and was markedly (*P* **<**0.05, versus the DSS group) restored by 40 mg/kg PAO treatment (**Figure 4B**). Beta diversity, analyzed by Principal Component Analysis (PCA) and Principal Co-ordinates Analysis (PCoA, including unweighted and weighted UniFrac distances), showed distinct separation in samples between Control and other groups, with the samples from 40 mg/kg PAO group clustering closer to the Control group (**Figures 4C–E**).

Taxonomic analysis revealed the composition of the gut microbiota community across various taxonomic levels—phylum, class, order, family, genus, and species—in samples from the Control, DSS, and PAO (40 mg/kg) groups. A total of 15 phyla were consistently identified across all groups (**Figure 4F**). Notably, *Bacteroidetes*, *Verrucomicrobia* and *Firmicutes* accounted for over 90% of the total classified sequences at the phylum level (**Figure 4F**, **Table 5**). Compared to the DSS group, the Control group exhibited a significant decrease (*P*<0.05) in the relative abundance of *Verrucomicrobia*, *Epsilonbacteraeota*, *Deferribacteres*, and *Cyanobacteria*, and a marked increase (*P*<0.05) in *Bacteroidetes* and *Actinobacteria*. Following treatment with 40 mg/kg PAO, there was a dramatic decrease (*P*<0.05) in *Verrucomicrobia* and a significant increase (P<0.05) in *Firmicutes* in the UC mice (**Table 5**). Stacked species distribution histograms highlighted the top 29 most abundant species across the different taxonomic levels and groups (**Figure 4F**).

**Table 5:** The relative abundance of the most representative phylum in the gut of experimental mice.

	Taxonomic group	Relative abundance (%)			Variation
		Control	DSS	PAO (40 mg/kg)	
Phylum	*Bacteroidetes*	68.68 ± 8.44*	35.00 ± 5.49	39.27 ± 11.14	↓
	*Verrucomicrobia*	6.40 ± 3.59*	41.63 ± 9.63	22.61 ± 14.00*	↑
	*Firmicutes*	18.78 ± 4.16	13.59 ± 6.41	27.26 ± 8.61*	↓
	*Actinobacteria*	0.44 ± 0.35*	0.13 ± 0.03	0.22 ± 0.10	↓
	*Epsilonbacteraeota*	0.27 ± 0.29*	3.04 ± 4.14	1.97 ± 1.23	↑
	*Deferribacteres*	0.07 ± 0.05*	0.31 ± 0.24	0.89 ± 0.92	↑
	*Cyanobacteria*	0.01 ± 0.01*	0.11 ± 0.09	0.04 ± 0.03	↑

*P<0.05 indicates a statistically significant difference when compared with the DSS group (*n*=7).

The symbol ↑ refers to an increase in the relative abundance of colonic microbiota in the DSS group compared to the control group, and ↓ refers to a decrease in the relative abundance of colonic microbiota in the DSS group compared to the control group.

In the Control group, there was a significant increase (*P*<0.05) in the relative abundance of three classes (*Bacteroidales*, *Bacilli*, *Erysipelotrichia*), three orders (*Bacteroidales*, *Lactobacillales*, *Erysipelotrichales*), three families (Muribaculaceae, Peptococcaceae, *Erysipelotrichaceae*), one genera (*Muribaculaceae_unclassified*), and four species (*Muribaculaceae_unclassified*, *Lactobacillus_hilgardii*, *uncultured_ Clostridium_sp.*, *Anaerotignum_sp.*) compared to the DSS group. Conversely, three classes (*Deferribacteres*, *Campylobacteria*, *Verrucomicrobiae*), three orders (*Deferribacterales*, *Campylobacterales*, *Verrucomicrobiales*), six families (*Bacteroidaceae*, *Marinifilaceae*, *Rikenellaceae*, *Deferribacteraceae*, *Helicobacteraceae*, *Akkermansiaceae*), six genera (*Bacteroides*, *Odoribacter*, *Rikenellaceae_RC9_gut_group*, *Mucispirillum, Helicobacter*, *Akkermansia*), and six species (*Bacteroides_unclassified*, *Odoribacter_unclassified*, *Rikenellaceae_RC9_gut_group_unclassified*, *Mucispirillum_schaedleri*, *Helicobacter_unclassified*, *uncultured_Akkermansia_sp*.) were notably reduced (*P*<0.05) in the Control group relative to the DSS group (**Table 6**). Furthermore, in comparison to the DSS group, the relative abundance of two classes (*Clostridia*, *Firmicutes_unclassified*), two orders (*Clostridiales*, *Firmicutes_ unclassified*), five families (*Muribaculaceae*, *Clostridiaceae*, *Peptococcaceae*, *Ruminococcaceae*, *Firmicutes_unclassified*), four genera (*Muribaculaceae_unclassified*, *Clostridium*, *Lachnospiraceae_unclassified*, *Ruminococcaceae_UCG-014*), and four species (*Muribaculaceae_unclassified*, *uncultured_Clostridium_sp.*, *Anaerotignum_* sp., *Lachnospiraceae_unclassified*) exhibited a dramatic increase (*P*<0.05) in the 40 mg/kg PAO group. In contrast, one Class (*Verrucomicrobiae*), one order (*Verrucomicrobiales*), two families (*Bacteroidaceae*, *Akkermansiaceae*), two genera (*Bacteroides*, *Akkermansia*), and three species (*Bacteroides_unclassified*, *Ruminiclostridium_unclassified*, *uncultured_ Akkermansia_sp.*) were significantly decreased (*P*<0.05) in the 40 mg/kg PAO group relative to the DSS group (**Table 6**).

**Table 6:** Relative abundance of the most representative taxonomic levels in the gut of experimental mice.

Phylum	Taxonomic group	Category	Relative abundance (%)			Variation
			Control	DSS	PAO (40 mg/kg)	
*Actinobacteria*	*Actinobacteria*	class	68.66 ± 0.13	34.95 ± 0.01	39.22 ± 0.06	↓
	*Bifidobacteriales*	order	68.66 ± 0.13	34.95 ± 0.01	39.22 ± 0.06	↓
*Bacteroidetes*	*Bacteroidia*	class	68.66 ± 8.44*	34.95 ± 5.47	39.22 ± 11.12	↓
	*Bacteroidales*	order	68.66 ± 8.44*	34.95 ± 5.47	39.22 ± 11.12	↓
	*Bacteroidaceae*	family	0.80 ± 0.32*	7.19 ± 2.84	3.33 ± 2.52*	↑
	*Marinifilaceae*	family	0.32 ± 0.16*	3.56 ± 2.37	2.63 ± 1.99	↑
	*Muribaculaceae*	family	62.88 ± 7.83*	12.32 ± 4.27	23.97 ± 5.42*	↓
	*Rikenellaceae*	family	1.29 ± 0.56*	10.21 ± 6.24	5.73 ± 3.57	↑
	*Bacteroides*	genus	0.80 ± 0.32*	7.18 ± 2.84	3.33 ± 2.52*	↑
	*Odoribacter*	genus	0.32 ± 0.16*	3.56 ± 2.37	2.63 ± 1.99	↑
	*Muribaculaceae_unclassified*	genus	60.65 ± 7.80*	10.75 ± 4.27	22.93 ± 5.55*	↓
	*Rikenellaceae_RC9_gut_group*	genus	0.21 ± 0.14*	8.72 ± 5.69	4.20 ± 2.97	↑
	*Bacteroides_unclassified*	species	0.06 ± 0.04*	3.70 ± 2.70	0.76 ± 0.62*	↑
	*Odoribacter_unclassified*	species	0.32 ± 0.16*	3.56 ± 2.37	2.63 ± 1.99	↑
	*Muribaculaceae_unclassified*	species	60.65 ± 7.80*	10.75 ± 4.27	22.93 ± 5.55*	↓
	*Rikenellaceae_RC9_gut_group_unclassified*	species	0.21 ± 0.14*	8.72 ± 5.69	4.20 ± 2.97	↑
*Cyanobacteria*	*Melainabacteria*	class	0.01 ± 0.01	0.11 ± 0.10	0.04 ± 0.03	↓
	*Gastranaerophilales*	order	0.01 ± 0.01	0.11 ± 0.10	0.04 ± 0.03	↓
	*Gastranaerophilales_unclassified*	family	0.01 ± 0.01	0.11 ± 0.10	0.04 ± 0.03	↑
	*Gastranaerophilales_unclassified*	genus	0.01 ± 0.01	0.11 ± 0.10	0.04 ± 0.03	↑
	*Gastranaerophilales_unclassified*	species	0.01 ± 0.01	0.11 ± 0.10	0.04 ± 0.03	↑
*Deferribacteres*	*Deferribacteres*	class	0.07 ± 0.05*	0.31 ± 0.24	0.89 ± 0.92	↑
	*Deferribacterales*	order	0.07 ± 0.05*	0.31 ± 0.24	0.89 ± 0.92	↑
	*Deferribacteraceae*	family	0.07 ± 0.05*	0.31 ± 0.24	0.89 ± 0.92	↑
	*Mucispirillum*	genus	0.07 ± 0.05*	0.31 ± 0.24	0.89 ± 0.92	↑
	*Mucispirillum_schaedleri*	species	0.05 ± 0.04*	0.21 ± 0.16	0.58 ± 0.59	↑
*Proteobacteria*	*Campylobacteria*	class	0.27 ± 0.29*	3.04 ± 4.14	1.97 ± 1.23	↑
	*Campylobacterales*	order	0.27 ± 0.29*	3.04 ± 4.14	1.97 ± 1.23	↑
	*Helicobacteraceae*	family	0.27 ± 0.29*	3.04 ± 4.14	1.97 ± 1.23	↑
	*Helicobacter*	genus	0.27 ± 0.29*	3.04 ± 4.14	1.97 ± 1.23	↑
	*Helicobacter_unclassified*	species	0.27 ± 0.29*	3.04 ± 4.14	1.97 ± 1.23	↑
*Firmicutes*	*Bacilli*	class	2.44 ± 2.03*	0.50 ± 0.28	0.39 ± 0.30	↓
	*Clostridia*	class	13.87 ± 4.64	12.47 ± 6.29	25.80 ± 8.75*	↓
	*Erysipelotrichia*	class	1.95 ± 2.28*	0.29 ± 0.15	0.44 ± 0.40	↓
	*Firmicutes_unclassified*	class	0.51 ± 0.28	0.31 ± 0.16	0.61 ± 0.28*	↓
	*Lactobacillales*	order	2.31 ± 2.01*	0.45 ± 0.23	0.38 ± 0.30	↓
	*Clostridiales*	order	13.87 ± 4.64	12.47 ± 6.29	25.80 ± 8.75*	↓
	*Erysipelotrichales*	order	1.95 ± 2.28*	0.29 ± 0.15	0.44 ± 0.40	↓
	*Firmicutes_unclassified*	order	0.51 ± 0.28	0.31 ± 0.16	0.61 ± 0.28*	↓
	*Clostridiaceae*	family	0.64 ± 0.50	0.22 ± 0.23	0.48 ± 0.25*	↓
	*Clostridiales_unclassified*	family	1.14 ± 0.64	0.76 ± 0.82	1.90 ± 2.28	↓
	*Lachnospiraceae*	family	6.08 ± 3.18	6.01 ± 4.75	7.92 ± 4.01	↓
	*Peptococcaceae*	family	0.11 ± 0.08*	0.02 ± 0.02	0.24 ± 0.15*	↓
	*Ruminococcaceae*	family	5.32 ± 1.36	4.21 ± 1.02	13.03 ± 9.99*	↓
	*Erysipelotrichaceae*	family	1.95 ± 2.28*	0.29 ± 0.15	0.44 ± 0.40	↓
	*Firmicutes_unclassified*	family	0.51 ± 0.28	0.31 ± 0.16	0.61 ± 0.28*	↓
	*Clostridium*	genus	0.64 ± 0.50	0.22 ± 0.23	0.48 ± 0.25*	↓
	*Clostridiales_unclassified*	genus	1.14 ± 0.64	0.76 ± 0.82	1.90 ± 2.28	↓
	*Lachnospiraceae_unclassified*	genus	1.10 ± 0.82	0.44 ± 0.46	1.44 ± 0.46*	↓
	*Ruminiclostridium*	genus	0.25 ± 0.18	0.91 ± 0.69	0.40 ± 0.30	↑
	*Ruminiclostridium_9*	genus	0.83 ± 0.51	0.36 ± 0.25	1.04 ± 0.99	↓
	*Ruminococcaceae_UCG-014*	genus	1.72 ± 1.02	1.00 ± 0.67	7.70 ± 9.09*	↓
	*Lactobacillus_hilgardii*	species	0.86 ± 0.80*	0.00 ± 0.00	0.18 ± 0.16	↓
	*uncultured_Clostridium_sp.*	species	0.48 ± 0.41*	0.07 ± 0.07	0.28 ± 0.15*	↓
	*Clostridiales_unclassified*	species	1.14 ± 0.64	0.76 ± 0.82	1.90 ± 2.28	↓
	*Anaerotignum_sp.*	species	0.15 ± 0.08*	0.13 ± 0.07	0.34 ± 0.28*	↓
	*Lachnospiraceae_unclassified*	species	1.10 ± 0.82	0.44 ± 0.46	1.44 ± 0.46*	↓
	*Ruminiclostridium_9_unclassified*	species	0.83 ± 0.51	0.36 ± 0.25	1.04 ± 0.99	↓
	*Ruminiclostridium_unclassified*	species	0.25 ± 0.18	0.91 ± 0.69	0.40 ± 0.30*	↑
	*Ruminococcaceae_unclassified*	species	0.14 ± 0.10	0.06 ± 0.03	0.24 ± 14.00	↓
*Verrucomicrobia*	*Verrucomicrobiae*	class	6.40 ± 3.59*	40.88 ± 9.63	22.61 ± 0.30*	↑
	*Verrucomicrobiales*	order	6.40 ± 3.59*	40.88 ± 9.63	22.61 ± 0.30*	↑
	*Akkermansiaceae*	family	6.40 ± 3.59*	40.88 ± 9.63	22.61 ± 0.30*	↑
	*Akkermansia*	genus	6.40 ± 3.59*	40.88 ± 9.63	22.61 ± 0.30*	↑
	*uncultured_Akkermansia_sp.*	species	6.40 ± 3.59*	40.86 ± 9.63	22.59 ± 14.00*	↑

*P<0.05 indicates a statistically significant difference when compared with DSS group (*n*=7).

The symbol ↑ refers to an increase in the relative abundance of colonic microbiota in the DSS group compared to the control group, and ↓ refers to a decrease in the relative abundance of colonic microbiota in the DSS group compared to the control group.

### PAO reduced the abundance of characteristic colonic flora in UC mice

**Figure 5:**
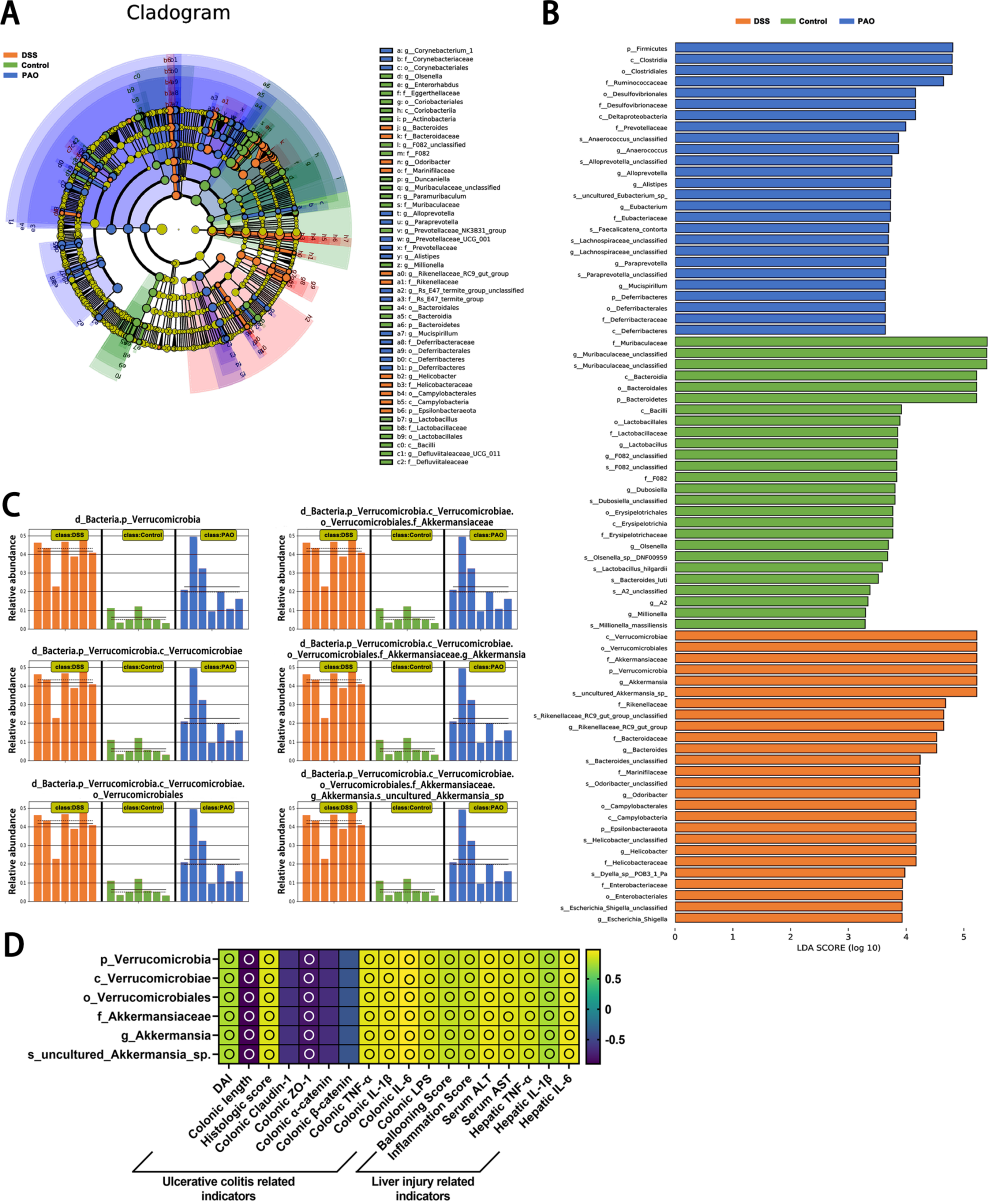
Linear discriminant analysis Effect Size (LEfSe) of microbiota composition (*n*=7). **(A)** Comprehensive depiction of LEfSe analysis presented as a cladogram. **(B)** Results of LDA scores for specifically enriched bacterial taxa within the gut microbiota of each group, where LDA score threshold >3 and *P*<0.05. **(C)** All-against-all algorithmic comparison using LDA in conjunction with LEfSe. **(D)** Spearman correlation analysis of the relationship between the DSS group's unique colonic microbiota and ulcerative colitis and liver injury-related indicators, displayed as a heatmap: an empty circle signifies |r|>0.6 and *P*<0.01.

Distinct gut microbiota taxa were discerned among the groups using Linear Discriminant Analysis Effect Size (LEfSe), revealing significant inter-group differences with an LDA score threshold of more than 3. In the Control group, 26 taxa from the phyla *Bacteroidetes* and *Actinobacteria* were predominant. The DSS group was characterized by 26 taxa from the phyla *Verrucomicrobia* and *Epsilonbacteraeota*; and the 40 mg/kg PAO group showed an abundance of 26 taxa from the phyla *Firmicutes* and *Deferribacteres* (**Figures 5A, B**). Notably, the *Firmicutes* phylum was particularly influential in the 40 mg/kg PAO group, with an LDA score exceeding 4.5, whereas *Bacteroidetes* was the dominant flora in the Control group, with an LDA score above 5.0. Additionally, the *Verrucomicrobia* phylum was identified as a distinctive gut microbiota signature in the DSS group, with an LDA score surpassing 5.0 (**Figure 5B**).

The LEfSe all-against-all algorithm substantiated that, compared to other groups, the DSS group had a significant increase in taxa at various taxonomic levels: *Verrucomicrobia* at the phylum level, *Verrucomicrobiae* at the class level, *Verrucomicrobiales* at the order level, *Akkermansiaceae* at the family level, *Akkermansia* at the genus level, and *uncultured_Akkermansia_sp* at the species level (**Figure 5C**). Spearman correlation analysis revealed positive associations (|r|>0.6, all *P*<0.05) between *Verrucomicrobia*, *Verrucomicrobiae*, *Verrucomicrobiales*, *Akkermansiaceae*, *Akkermansia*, and *uncultured_Akkermansia_sp* and biomarkers related to ulcerative colitis and liver injury, except for the correlation involving colonic claudin-1, α-catenin, and β-catenin (**Figure 5D**). Of which, the correlation between these microbial groups and colonic length, claudin-1, ZO-1, α-catenin, and β-catenin was inversely related to the associations observed with other indicators.

### PAO ameliorated colonic metabolic dysregulation in UC mice

**Figure 6:**
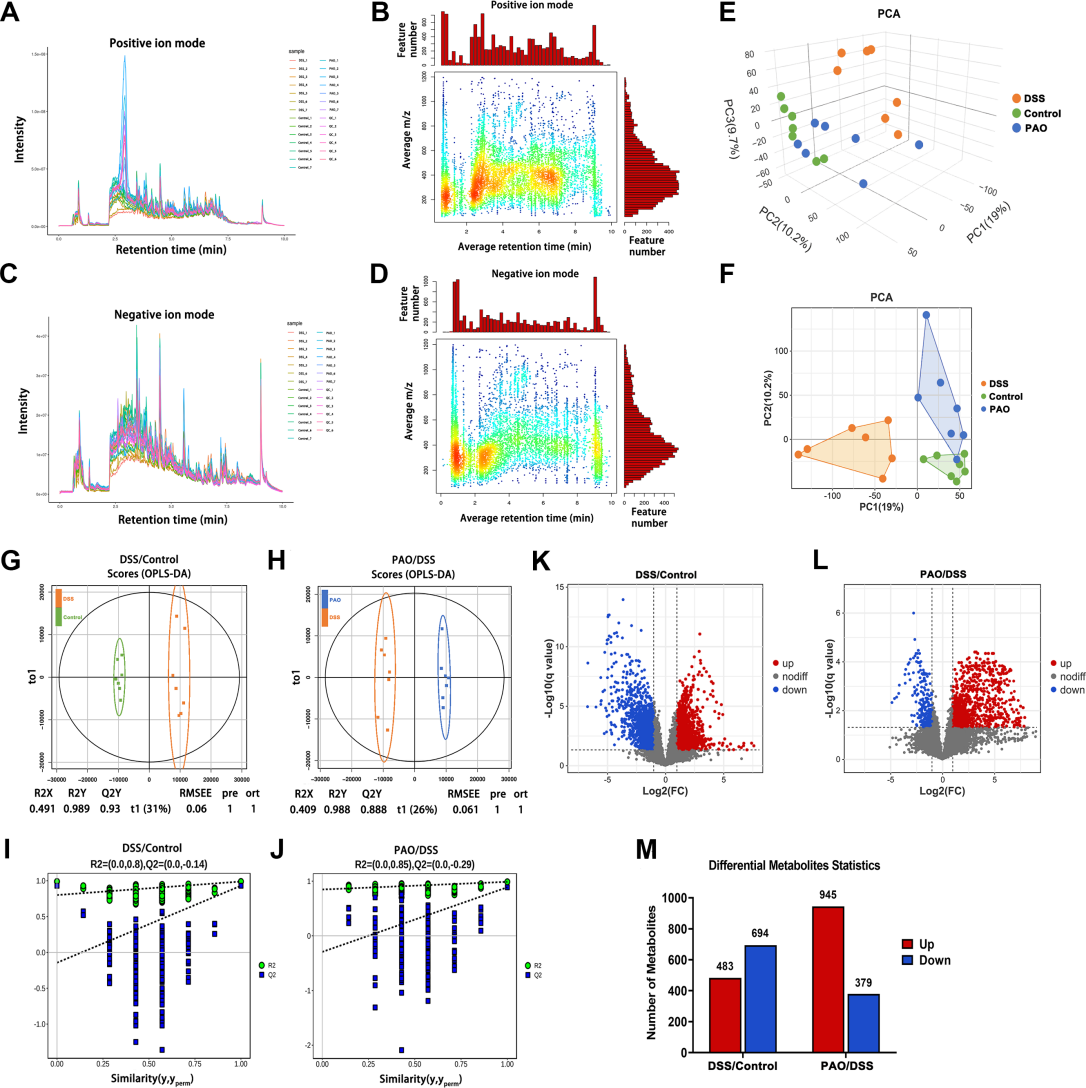
Metabolomic analysis (*n*=7). **(A)** Total ion current and **(B)** m/z-Retention Time (RT) distribution of metabolites in positive ion mode. **(C)** Total ion current and **(D)** m/z-RT distribution of metabolites in negative ion mode. **(E)** Three-dimensional (3D) and **(F)** Two-dimensional (2D) Principal Component Analysis (PCA) score plots. **(G)** OPLS-DA score plot of metabolites differentiating the DSS and Control groups. **(H)** OPLS-DA score of metabolites between the PAO and DSS group. **(I)** Permutation tests of the OPLS-DA model for the DSS versus Control group comparison. **(J)** Permutation tests of the OPLS-DA model for the PAO versus DSS group comparison. **(K)** Volcano plot illustrating the significance analysis of metabolites when comparing the DSS and Control groups. **(L)** Volcano plot illustrating the significance analysis of metabolites when comparing the PAO and DSS groups. **(M)** Quantification of differential metabolites between the DSS and Control groups, as well as between the PAO and DSS groups, respectively.

Colonic metabolic dysregulation plays a pivotal role in the pathogenesis of colitis. To explore the metabolic shifts in the colon of UC mice and the effect of PAO treatment on these alterations, an extensive untargeted metabolomic analysis was conducted using UPLC-MS/MS. The mass-to-charge ratio (m/z) of positive ions was predominantly found between 100 and 590, with retention times clustering between 2.2 and 3.0 minutes (**Figures 6A, B**). For negative ions, the m/z range was 150 to 450, and retention times were concentrated between 0.6 and 1.4 minutes (**Figures 6C, D**).

Two-dimensional and three-dimensional PCA score plots revealed distinct metabolic profiles, with samples from the DSS group being metabolically separated from other groups. Remarkably, the 40 mg/kg PAO and Control groups exhibited closely related metabolic patterns (**Figures 6E, F**). Orthogonal Partial Least Squares-Discriminant Analysis (OPLS-DA) was employed to statistically dissect the metabolomic variance among different groups. A clear distinction was observed between the DSS and Control groups (R2Y=0.989, Q2Y=0.930), with complete separation of metabolites along the t1 dimension (**Figure 6G**). Similarly, a pronounced separation was noted between the 40 mg/kg PAO and DSS groups (R2Y=0.988, Q2Y=0.888), with distinct metabolite clustering in the t1 dimension (**Figure 6H**). A permutation test was conducted to ascertain the robustness of the PLS-DA models. Both the DSS versus Control and the 40 mg/kg PAO versus DSS models demonstrated reduced R2 and Q2 values compared to their original states, with Q2 values approaching zero, suggesting that these models had not been over-fitted (**Figures 6I, J**).

Subsequent differential metabolite screening was performed based on these validated OPLS-DA models. A comparative analysis of metabolites between the DSS and Control groups identified a total of 1177 metabolites, with 483 being significantly upregulated and 694 significantly downregulated. In contrast, the comparison between the 40 mg/kg PAO and DSS groups revealed 1324 identified metabolites, among which 945 were markedly upregulated, and 379 were markedly downregulated (**Figures 6K–M**).

### PAO alleviated colitis and liver injury by enhancing colonic purine metabolism in UC mice

**Figure 7:**
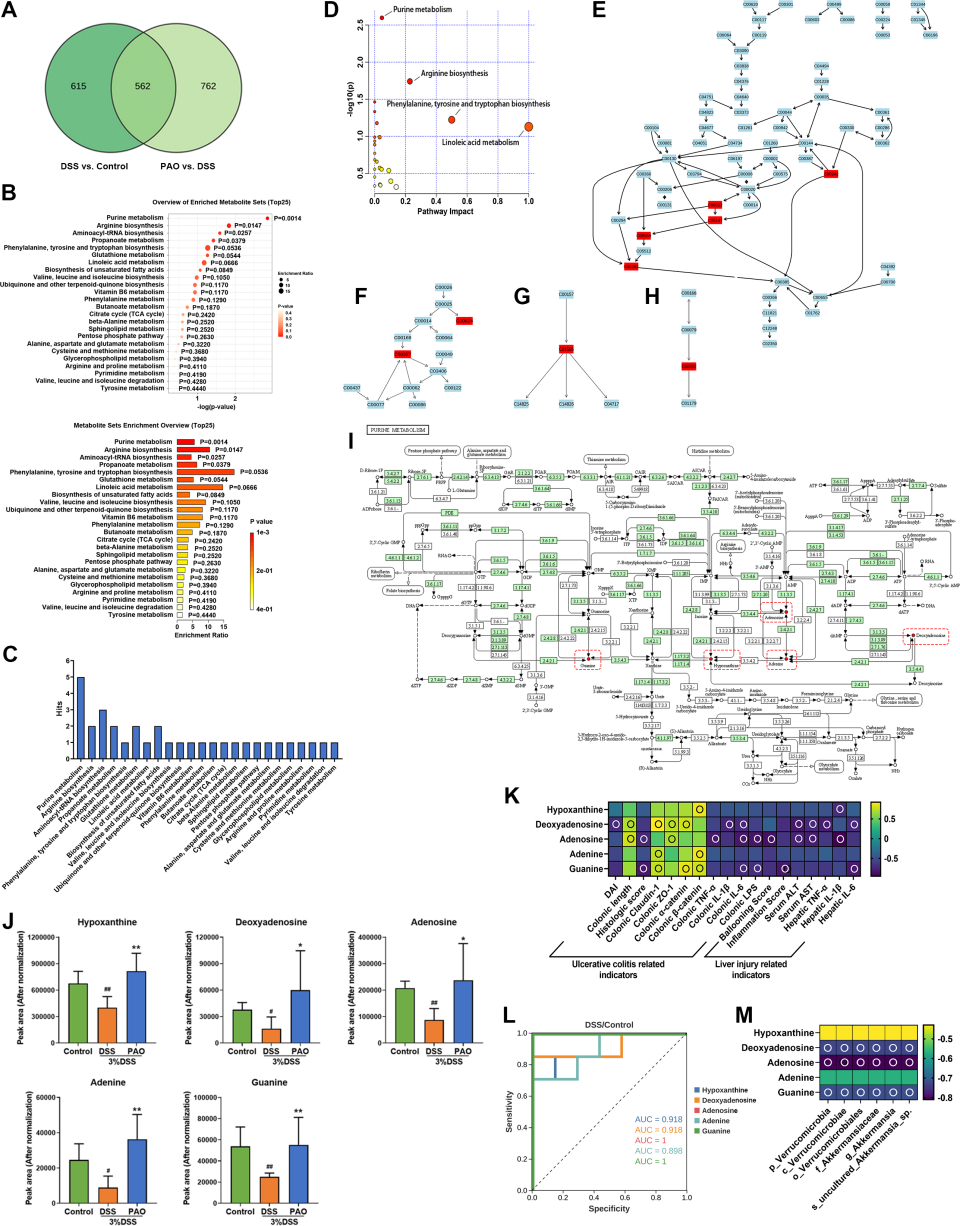
Enrichment and pathway analysis of common differential metabolites (*n*=7). **(A)** Venn diagram illustrating the overlap of differential metabolites. **(B)** Enrichment analysis: Pathways significantly associated with the common differential metabolites, with *P*-values indicating a statistically meaningful correlation. **(C)** Distribution of common differential metabolites in the pathways obtained by enrichment analysis. Pathway analysis: **(D)** Metabolic pathway enrichment bubble plot: It represents matched pathways based on *P*-values from enrichment analysis (y-axis) and impact values from topology analysis (x-axis). The color and size of each circle correspond to *P*-value significance and pathway impact, respectively. Notable disruptions in metabolism are indicated by smaller *P*-values and larger circles. The top 4 pathways are identified based on *P*-value significance. **(E)** Representation of common differential metabolites in the Purine metabolism pathway, highlighted in red. **(F)** Representation of common differential metabolites in Arginine biosynthesis, highlighted in red. **(G)** Representation of common differential metabolites in Linoleic acid metabolism, highlighted in red. **(H)** Representation of common differential metabolites in Phenylalanine, tyrosine, and tryptophan biosynthesis, highlighted in red. **(I)** Map of Purine metabolism from the KEGG database: Red dots and boxes indicate metabolites enriched in this pathway. **(J)** Quantification of the relative abundance (normalized peak area) of hypoxanthine, deoxyadenosine, adenosine, adenine, and guanine. **(K)** Spearman correlation analysis presented as a heatmap, illustrating the relationship between hypoxanthine, deoxyadenosine, adenosine, adenine, guanine, and ulcerative colitis and liver injury-related indicators: an empty circle denotes |r|>0.6 and *P*<0.01. **(L)** Receiver Operating Characteristic (ROC) curve analysis to differentiate between the DSS and Control groups based on the metabolite abundance of hypoxanthine, deoxyadenosine, adenosine, adenine, and guanine. **(M)** Spearman correlation analysis presented as a heatmap, depicting the relationship between hypoxanthine, deoxyadenosine, adenosine, adenine, guanine, and specific colonic microbiota of the DSS group: hollow circle indicates |r|>0.6 and *P*<0.01.

To discern the effect of PAO on the colonic metabolic profile of UC mice, we conducted a comparative analysis focusing on the differential metabolites identified through the OPLS-DA model. A Venn diagram revealed a shared set of 562 differential metabolites between the DSS versus Control group and the 40 mg/kg PAO versus DSS group models (**Figure 7A**). Subsequent enrichment analysis indicated that these common differential metabolites were significantly enriched in four metabolic pathways (*P<0.05*), specifically Purine metabolism, Arginine biosynthesis, Aminoacyl-tRNA biosynthesis, and Propanoate metabolism (**Figure 7B**). Notably, Purine metabolism stood out with the highest number of associated common differential metabolites, totaling five hits (**Figure 7C**), and it was the only pathway with a *P*-value < 0.01 in the enrichment analysis (**Figure 7B**).

Pathway analysis confirmed that alterations in Purine metabolism, Arginine biosynthesis, Phenylalanine, tyrosine and tryptophan biosynthesis, and Linoleic acid metabolism were implicated in the colonic metabolic shifts (**Figure 7D**). Further investigation highlighted Purine metabolism as involving the most significant number of common differential metabolites (**Figures 7E–H**). This convergence of evidence suggests that Purine metabolism is a pivotal pathway influencing the progression of UC and the therapeutic effects of PAO. The metabolites within this pathway included hypoxanthine, deoxyadenosine, adenosine, adenine, and guanine (**Figure 7I**). Compared to the Control group, the levels of these metabolites were significantly (*P*<0.05) decreased in the DSS group. However, treatment with 40 mg/kg PAO led to a marked increase (*P*<0.05) in the levels of these purine metabolites compared to the DSS group (**Figure 7J**), indicating that PAO promotes colonic purine metabolism in UC mice.

Spearman correlation analysis was conducted to explore the relationship between purine metabolites and UC and liver injury-related biomarkers (**Figure 7K**). Hypoxanthine showed a positive correlation with colonic β-catenin and an inverse correlation with hepatic IL-1β (|r|>0.6, all P<0.05). Deoxyadenosine was positively correlated with colonic length, claudin-1, ZO-1, α-catenin, and negatively with DAI, colonic IL-1β and IL-6, serum ALT and AST, hepatic TNF-α and IL-6 (|r|>0.6, all P<0.05). Adenosine was positively associated with colonic length, ZO-1, and inversely related to histologic score, colonic TNF-α, IL-6 and LPS, Ballooning score, serum ALT and AST, and hepatic IL-1β (|r|>0.6, all P<0.05). Adenine presented a specific positive correlation with colonic claudin-1 and β-catenin (|r|>0.6, all P<0.05). Guanine was positively linked to colonic claudin-1, α-catenin and β-catenin, and negatively related to histologic score, colonic IL-6 and LPS, inflammation score, and hepatic IL-6 (|r|>0.6, all P<0.05).

Receiver operating characteristic curve (ROC) analysis revealed that the levels of hypoxanthine, deoxyadenosine, adenosine, and guanine could effectively distinguish between the DSS and Control groups, with AUC values exceeding 0.9, except for adenine, which had an AUC of 0.8988 (**Figure 7L**). These findings suggest that hypoxanthine, deoxyadenosine, adenosine, and guanine may serve as potential biomarkers for UC. Furthermore, Spearman correlation analysis indicated that the levels of deoxyadenosine, adenosine, and guanine were inversely (|r|>0.6, all P<0.05) related to the abundances of *Verrucomicrobia*, *Verrucomicrobiae*, *Verrucomicrobiales*, *Akkermansiaceae*, *Akkermansia* and *uncultured_Akkermansia_sp* (**Figure 7M**).

## Discussion

This study has shown that patchoulene epoxide (PAO) significantly alleviates ulcerative colitis (UC) and liver injury. The treatment’s effectiveness is demonstrated by reduced Disease Activity Index (DAI) and histological pathology scores, alongside improvements in colon length and a decrease in colonic and hepatic inflammation. Moreover, PAO modulates the balance of the colonic microbiota, which is integral to its ameliorative effects on colonic and hepatic damage. The restoration of microbial balance by PAO enhances colonic purine metabolism to amplify its beneficial effects on the colon and liver. It provides substantial evidence that the restoration of colonic flora equilibrium and metabolic regulation are key mechanisms by which PAO exerts its protective effects against colitis and liver injury.

Colonic barrier dysfunction is pivotal in the pathogenesis of UC. Tight junctions (TJs) and adherens junctions (AJs) are critical components of this barrier, with ZO-1 and Claudin-1 being essential for TJ formation and stabilization. AJs, which include proteins like α-catenin and β-catenin, are vital for cell adhesion and actin cytoskeleton regulation (26, 27). Consistent with previous research (28, 29), our study observed a disruption in the colonic barrier of UC mice, characterized by diminished expressions of TJ and AJ-related genes (ZO-1, Claudin-1, α-catenin and β-catenin). PAO treatment was found to bolster these gene expressions, thereby strengthening the colonic barrier.

Colonic barrier disruption is often linked to elevated levels of pro-inflammatory cytokines and lipopolysaccharide (LPS). Inflammation is a hallmark of UC, with increased levels of cytokines such as TNF-α, IL-1β, and IL-6 fostering mucosal infiltration and inflammation, leading to colonic barrier damage (30–32). LPS, an endotoxin derived from Gram-negative bacteria, triggers inflammatory responses and tissue damage (33). When the colonic barrier is compromised, colonic permeability increases, allowing pro-inflammatory factors and bacterial endotoxins to translocate to the liver (34). These gut-derived pro-inflammatory factors and bacterial endotoxins induce liver injury accompanied by elevated levels of serum ALT, AST and hepatic pro-inflammatory factors (35–37). This study confirmed that the liver injury of UC mice is closely related to LPS levels in the colons and serum (**Figure 3K**). Moreover, the relationship between the them still needs to be further studied by designing more comprehensive experiments in the future.

Gut microbiota disturbances are intimately connected to UC-related colonic and hepatic damage (38). Flora imbalance first manifests as changes in microbial diversity and relative abundance (39). In this study, a reduction in microbial diversity and alterations in community structure were observed in UC mice. Notably, specific bacterial groups including *Verrucomicrobia*, *Verrucomicrobiae*, *Verrucomicrobiales*, *Akkermansiaceae*, *Akkermansia* and *uncultured_Akkermansia_sp* were over-represented in the colonic flora of UC mice (**Figures 4**, **5**). PAO significantly reduced the abundance of these microbiotas. Interestingly, PAO promotes a shift towards a microbiota composition resembling that of healthy mice, dominated by *Muribaculaceae*. As the genus level provides a better reflection of changes in microbial ecological niches, an analysis of the colonic flora composition based on the genus level classification is warranted (40). This study illustrated that the colonic flora homeostasis was dominated by *Muribaculaceae* in healthy mice (**Figure 4F**, **Table 6**). Upon the onset of colitis and liver injury in mice, the abundance of *Muribaculaceae* sharply decreased, and *Akkermansia* becoming the dominant species. *Akkermansia*, known for its mucin-degrading capabilities, is markedly increased in DSS-treated mice, contributing to heightened inflammation (41, 42). This study’s correlation analysis also indicates a strong link between *Akkermansia* overgrowth and the colonic and hepatic inflammatory response (**Figure 5D**). However, PAO treatment effectively controls the proliferation of *Akkermansia*, restoring the microbiota composition to a state similar to that of healthy mice, where *Muribaculacea* is predominant (**Figure 4F**, **Table 6**). What deserves our further consideration is that many studies have shown that *Akkermansia* can be used to treat UC (43, 44), so the complex role of *Akkermansia* in UC requires further elucidation, particularly in the context of clinical UC.

Colonic metabolic dysregulation is a principal contributor to colon and liver injury in UC, a condition exacerbated by an imbalance in the colonic microbiota. Increasing studies have shown that metabolic disorders in the colon are pivotal in the progression of UC (45). In this context, the analysis of differential metabolites in the colonic content of UC mice, as part of a metabonomic study, is of particular significance. In this study, we utilized the OPLS-DA model to scrutinize the differential metabolites resulting from the DSS-Control comparison and the PAO-DSS comparison (**Figure 6**). To deepen our understanding of PAO’s impact on the colonic metabolism of UC mice, we conducted a commonality analysis on the significant differential metabolites identified from the two models (**Figure 7A**). The metabolite comparison between the DSS and Control groups primarily aimed to identify DSS biomarkers, while the comparison between the PAO and DSS groups offered a broader metabolic overview, potentially revealing additional metabolites with significantly altered abundances. The identified biomarkers are closely associated with DSS development, rendering them a more precise and potent basis for evaluating drug efficacy. Through enrichment and pathway analyses of the common differential metabolites (**Figures 7B–H**), we discovered that purine metabolism is a critical pathway in the advancement of DSS and the anti-DSS effectiveness of PAO. Metabolites within the purine metabolism pathway, including hypoxanthine, deoxyadenosine, adenosine, and guanine, were identified as potential biomarkers for UC (**Figure 7L**). A wealth of studies has underscored the importance of purine metabolism regulation in the regeneration of the colonic barrier during UC development (46, 47), this finding is further substantiated by the correlation analysis in this study (**Figure 7K**).

This study revealed that the imbalance in purine metabolism in UC mice was primarily characterized by a markedly reduced abundance of hypoxanthine, deoxyadenosine, adenosine, adenine, and guanine, a change that PAO treatment significantly counteracted (**Figure 7J**). Prior research has predominantly focused on the role of purine metabolism in colonic regeneration in UC, with less emphasis on its role in UC-related liver injury. This study intriguingly established that purine metabolism regulation is also intimately connected to the repair of UC-related liver injury, with the correlation analysis indicating significant associations between hypoxanthine, deoxyadenosine, adenosine, and guanine and liver injury-related indicators. This suggests that these metabolites are not only potential biomarkers for UC-related colonic injury but also UC-related liver injury, although future clinical studies are necessary to validate these findings. The study further discovered that deoxyadenosine, adenosine, and guanine in the purine metabolism pathway were significantly negatively correlated with the characteristic colonic flora of UC (**Figure 7M**). This implies that the overabundance of *Verrucomicrobia*, *Verrucomicrobiae*, *Verrucomicrobiales*, *Akkermansiaceae*, *Akkermansia* and *uncultured_Akkermansia_sp* could indeed disrupt purine metabolism in the colon of UC mice. Gut microbiota has been identified as a key regulator of purine homeostasis in the host. Through modulation of purine metabolism, gut microbiota may have a profound impact on host health, including influencing the pathogenesis of ulcerative colitis (UC) (48, 49). In this study, we found that *Akkermansia* is predominant in the gut bacteria of UC mice. *Akkermansia* has an inhibitory effect on purine metabolism (50), and consistent with previous research, purine metabolism was found to be suppressed in UC mice. Following PAO administration, the gut microbiota composition of UC mice shifted to a predominance of *Muribaculaceae*. However, it has been shown that there is no significant correlation between *Muribaculaceae* and purine metabolism (51). A more detailed analysis of the changes in the relative abundance of gut microbiota before and after PAO treatemnt revealed that *Akkermansia* showed the most significant change (**Table 6**). Therefore, we hypothesize that the regulation of purine metabolism by PAO may be associated with its reduction in the abundance of *Akkermansia*, and the underlying mechanism warrants further investigation. While preliminary evidence confirms that an imbalance in colonic flora can induce abnormal colonic metabolism in UC, the underlying mechanisms warrant further investigation. For instance, it remains to be elucidated whether the characteristic colonic flora of UC can induce abnormal metabolism through its functional enzymes.

## Conclusions

This study demonstrated the protective effect of PAO against colitis and liver injury through regulating colonic flora and purine metabolism, which highlights the holistic therapeutic potential of PAO in treating UC and associated organ injuries.

## Data Availability

The original contributions presented in the study are included in the article/supplementary material. Further inquiries can be directed to the corresponding authors.
